# Glanular Ischemia Following Circumcision: A Rare But Perilous Complication

**DOI:** 10.7759/cureus.69100

**Published:** 2024-09-10

**Authors:** Subash Kaushik TG, Nakul Aher, Rubina Singh, Hariharasudhan Sekar, Sriram Krishnamoorthy

**Affiliations:** 1 Urology, Sri Ramachandra Institute of Higher Education and Research, Chennai, IND

**Keywords:** circumcision, ischemia, necrosis, penis, pentoxifylline

## Abstract

Circumcision is a commonly performed surgical procedure both in childhood and in adults. Acute ischemia of the glans penis after circumcision is a rare but hazardous complication. If left untreated, it can lead to severe consequences such as blackish discolouration due to ischemia, necrosis, or rare organ loss. Herein, we report a case of a 30-year-old male who underwent circumcision for phimosis, following which, he developed ischemia of the glans penis that was managed with timely diagnosis, topical application of warm saline, and oral pentoxifylline. We report the need for a prompt diagnosis and early intervention and also highlight the possibility of dreadful sequelae if left undiagnosed or untreated.

## Introduction

Circumcision is a frequently performed urological procedure. It is an elective procedure performed by both skilled and unskilled professionals across the world, especially in the Islamic and Jewish populations. Though a simple and frequently performed surgery, the morbidity rate varies from 0.1% to 35% [1]. In one of the largest studies on 1,720 children undergoing circumcision, the overall complication rate was found to be 7.4% [2]. The complications, even if trivial, may appear serious, as the organ has functional and cosmetic importance. The common complications observed after circumcision include frenular bleeding, meatal stenosis, infection, and ventral skin discolouration. Inadequate or over-excision of the foreskin, especially done by registrars or junior residents, is another common but serious complication. Vascular complications following circumcision are rare. Such complications are commonly seen in diabetics with pre-existing vasculitis, most often related to compression bandages, continuous sutures leading to vascular compromise, trauma, or usage of vasoconstrictive agents for penile block [3-5]. Glanular ischemia following circumcision is one of the most dreaded complications. Partial or complete loss of the glans penis would inflict significant psychological damage to the patient, especially in the younger age group. The pathophysiology that leads to ischemia is still uncertain, but different studies suggest vasospasm of the glans after surgery, dorsal penile nerve block anaesthesia due to vascular obstruction, and hematoma after the procedure at the injection site, leading to ischemic endothelial injuries and necrosis of the glans as the common mechanisms [6]. For treating this condition, various methods such as hyperbaric oxygen therapy, topical testosterone application, intra-cavernosal glyceryl trinitrate injection, and low-dose heparin infusion have all been described in the literature [7]. The methylxanthine agent, pentoxifylline (PTX) is a potent vasodilator that enhances the micro-circulatory blood flow by reducing the blood viscosity and increasing the erythrocyte pliability, making it one of the first-choice agents to treat such conditions [8]. Here, we discuss a young male with glanular ischemia following circumcision and its management.

## Case presentation

**Figure 1:**
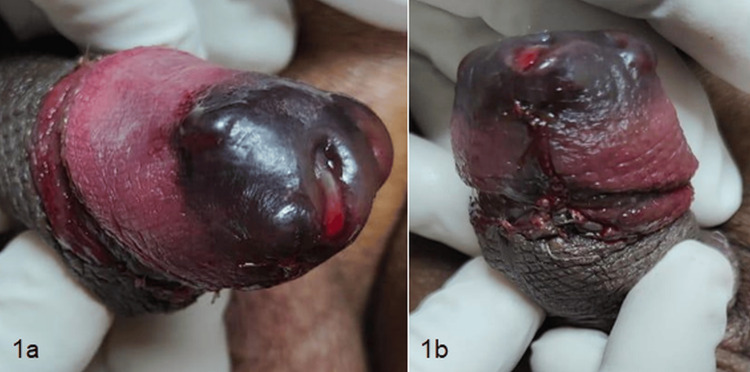
(a and 1) Oedematous glans penis with the tip showing blackish discolouration.

A 30-year-old non-diabetic male underwent elective circumcision for phimosis. Under general anaesthesia, supplemented with the penile block using 2% plain lignocaine, circumcision was done using the sleeve resection method. Haemostasis was obtained using interrupted sutures with 4-0 absorbable monofilament material. A self-adherent Coban dressing was done. The patient was discharged the same day, with the compression dressing in place. The next day morning, he presented with oedema with blackish discolouration of the tip of the glans penis (Figure 1).

**Figure 2:**
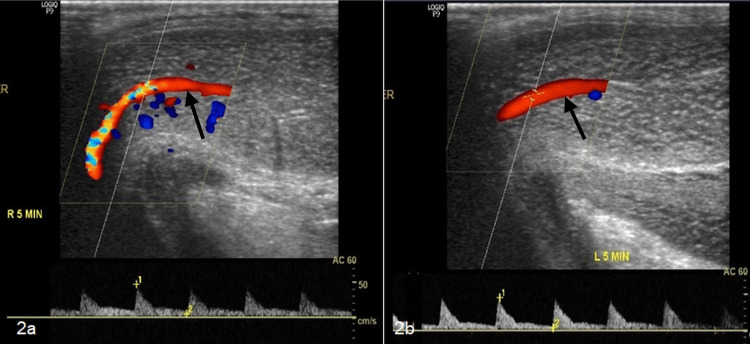
(a and b) Penile Doppler depicting normal blood flow in the dorsal penile vessels (black arrows) on both right and left sides supplying the corpora and glans penis, with no evidence of hematoma.

The rest of the glans penis was normal and showed blanching on pressure. No voiding difficulty was seen. Following this, a penile Doppler was performed, which revealed normal blood flow to the corpora cavernosa and corpora spongiosum (Figure 2).

**Figure 3:**
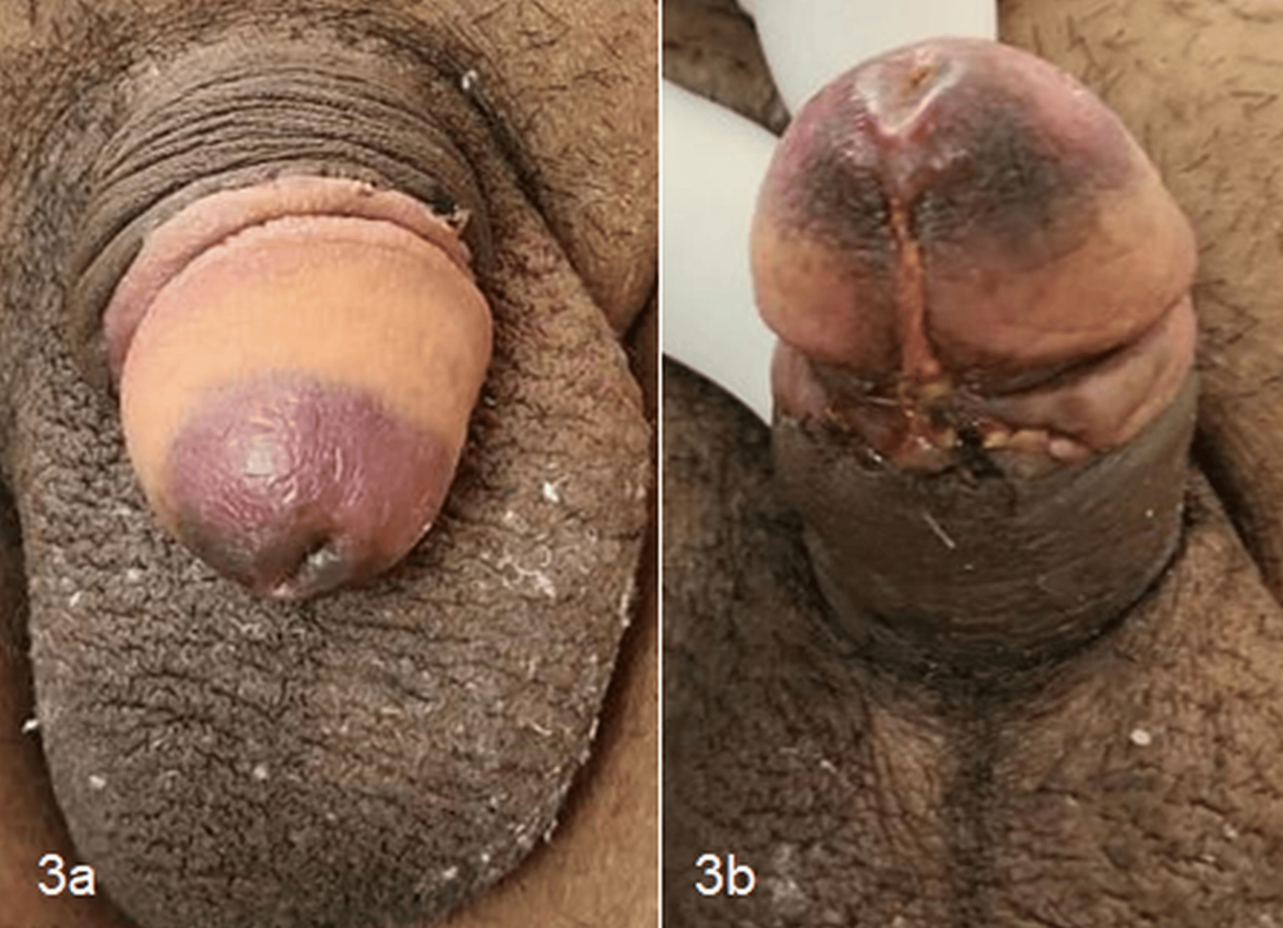
(a and b) Circumcision wound with the tip of the glans showing near normal pinkish colouration, which is regained after a week of commencement of pentoxifylline medication.

A diagnosis of glanular ischemia was made. A prompt decompression of the dressing was done as the immediate first step of the treatment. A possibility of a tight compression causing discolouration of the glans tip was considered, but as the rest of the proximal glans and the suture lines at the corona glandis were normal, this possibility, to a certain extent, was ruled out. The patient was started on 400 mg of oral pentoxifylline and warm saline dressings twice a day, which was maintained for seven days. After 48 hours of commencement of treatment, there was a gradual improvement in the discolouration, finally returning to near normal by the 10th postoperative day (Figure 3). At five weeks of follow-up, the appearance of the glans penis was normal with the preservation of sensation in the glans penis and normal morning erections.

## Discussion

Therapeutic circumcision is a commonly performed surgical procedure world over, by general surgeons and urologists. Religious neonatal circumcision is a commonly performed procedure by Muslims and Jews. Approximately, more than one-third of the males in this world are circumcised [9]. A recent study demonstrated that therapeutic circumcisions had the risk of complications twice that of non-therapeutic and religious ones (7.47% and 3.34%, respectively) [10]. The most common complications of circumcision were meatal stenosis and infections.

Glanular ischemia following circumcision is a rare complication. The potential causes of post-circumcision glanular ischemia are multifactorial. The diagnosis of glanular ischemia is more often clinical. The blackish discolouration of the tip of the glans penis was probably due to capillary ischemia, which is extremely difficult to diagnose using a Doppler probe. A normal blood flow into the corpora cavernosa and glans penis is often encouraging, as most often such patients show better reperfusion with vasodilators and supportive measures. If left unrecognised or untreated, the glans tip may go in for irreversible necrosis. For such cases, where the medical therapy fails or if irreversible necrosis sets in the glans, amputation can be performed as a surgical intervention [11]. However, we need to remember that the blood flow across the main vessels (as documented by normal blood flow) does not guarantee an adequate/optimal blood flow across the tiny capillaries of the tip of the glans penis. More often, the blackish discolouration at the tip of the glans may be the harbinger of an underlying vascular compromise, which should forewarn the treating physician to be proactive and initiate immediate appropriate measures.

While glanular ischemia is a distinct clinical diagnosis, authors would also like to highlight other potential complications such as infection or hematoma at the tip of the glans, which can also closely mimic glanular ischemia at the initial stages. Available literature sources suggest blood-vessel binding, excessive use of monopolar cautery, Dorsal penile block using bupivacaine with or without vasoconstrictor, and application of compression dressings as probable causes [12].

Various treatment strategies were reported in the literature with all of them of a common objective to induce vasodilation thereby promoting arterial blood flow and enhancing venous drainage, thus encouraging adequate revascularization of the ischemic glans. Ischemia, if not treated, may lead to necrosis and loss of part of the penis.

Extensive sporadic reporting of this complication has been published in the medical literature. Hyperbaric oxygen therapy, pentoxifylline, enoxaparin, sildenafil citrate, prostaglandin I2, corticosteroids, topical androgen, and nitroglycerine have all been described [13]. Some studies showed positive results with pentoxifylline (PTX), alone or along with other supportive measures such as warm saline dressings. The effect of pentoxifylline on ischemia-reperfusion injury might be linked to enhanced prostaglandins and inhibition of phosphodiesterase activity that stimulates cyclic AMP synthesis [14]. The learning points from this case scenario are as follows: i) A high index of clinical suspicion is mandatory to make a prompt diagnosis of this potentially reversible complication of circumcision. ii) Patients with peripheral vascular disease should be closely watched in the immediate postoperative period for the first 24 hours. iii) It is prudent to keep the glans tip open during dressings, which would enable an early identification of this complication.

## Conclusions

Glanular ischemia after circumcision may be due to multiple factors. A favourable outcome is reported in most cases. Unfavourable outcomes might either be due to delayed diagnosis or lack of awareness amongst patients and/or treating physicians. It is judicious to keep the tip of the glans uncovered when a patient is getting discharged from the hospital. Regular follow-up after discharge is mandatory to determine the therapeutic success. A high index of clinical suspicion, early diagnosis, and timely intervention would be organ-saving.
